# Identification of HBV-MLL4 Integration and Its Molecular Basis in Chinese Hepatocellular Carcinoma

**DOI:** 10.1371/journal.pone.0123175

**Published:** 2015-04-22

**Authors:** Hua Dong, Lan Zhang, Ziliang Qian, Xuehua Zhu, Guanshan Zhu, Yunqin Chen, Xiaoying Xie, Qinghai Ye, Jie Zang, Zhenggang Ren, Qunsheng Ji

**Affiliations:** 1 AstraZeneca Asian and Emerging market iMed, Zhangjiang Hi-Tech Park, Shanghai, P. R. China; 2 Liver Cancer Institute, Zhongshan Hospital, Key Laboratory of Carcinogenesis and Cancer Invasion, Ministry of Education, Fudan University, Shanghai, P. R. China; 3 R&D Information, AstraZeneca, Shanghai, P. R. China

## Abstract

To gain molecular insights of HBV integration that may contribute to HCC tumorigenesis, we performed whole transcriptome sequencing and whole genome copy number profiling of hepatocellular carcinoma (HCC) samples from 50 Chinese patients. We identified a total of 33 HBV-human integration sites in 16 of 44 HBV-positive HCC tissues, which were enriched in HBV genotype C-infected patients. In addition, significantly recurrent HBV-MLL4 integration (18%; 8/44) was found in this cohort of patients. Using long-range PCR and Sanger sequencing, we comprehensively characterized gDNA and cDNA sequences that encode for the HBV-MLL4 transcripts, and we revealed that HBV integration into *MLL4* exons led to much higher mRNA expression of *MLL4* than the integration into *MLL4* introns due to an alternative splicing mechanism. Moreover, the HBV-MLL4 integration occurred almost exclusively in *CTNNB1* and *TP53* wild-type patients. The integration was also associated with a distinct gene expression profile. In conclusion, this is the first report on the molecular basis of the *MLL4* integration driving *MLL4* over-expression. HBV-MLL4 integration occurred frequently in Chinese HCC patients, representing a unique molecular segment for HCC with HBV infection.

## Introduction

Hepatocellular carcinoma (HCC), which accounts for more than 80% of cases of primary liver cancer, is the third most common cause of cancer-related deaths globally [1]. Several risk factors are linked to HCC pathogenesis: alcoholic liver disease, chronic hepatitis B and C virus (HBV and HCV) infection, rare genetic disorders, and diabetes/obesity [1–5]. In China, the most common cause of HCC is chronic HBV infection, responsible for more than 50% of all cases [6]. HBV is phylogenetically classified into eight distinct genotypes (A–H), and genotype C is the most prevalent in the Chinese population [7]. Lines of evidence have supported the relationship between HBV infection and HCC. The risk for developing HCC among carriers of HBV surface antigen (HBsAg) has been proven to be 100 times greater than for those without the antigen [8], and 0.8 ~ 10% of chronic HBV carriers eventually develop HCC [9,10]. Furthermore, HBV vaccinations have significantly blocked HBV infections, which in turn have lowered the incidence of HCC [11,12].

The HBV genome encodes several viral proteins that are essential for its life cycle, including reverse transcriptase/DNA polymerase (pol), a capsid protein known as hepatitis B core antigen (HBcAg), and three envelope proteins (L, M, and S), which are functionally associated with the endoplasmic reticulum (ER) membrane during the virus replication process. The HBV genome also encodes protein x (HBx), which has been highly suggested to be a hepatocarcinogenic factor; about 90% of HBx transgenic mice developed HCC in one study [13]. In addition, transcription of the four open reading frames (ORFs) is controlled by four promoter elements (preS1, preS2, core/precore, and x) and two enhancer elements (Enh I and Enh II) [14]. However, the underlying mechanism for how HBV causes HCC has not yet been fully elucidated. One of the potential mechanisms is that the HBV genome actively integrates into the human genome, which not only leads to the persistence of the virus, but also induces genetic alterations in the host cells [15]. For examples, the integration of HBx/HBsAg genes into human *TERT*, *CCNE1*, and *MLL4* has frequently been observed in HCC [16,17]; HBx-LINE integration was confirmed to promote the tumorigenicity of HCC via the activation of Wnt/β-catenin signaling [18]. Consequently, several key questions are raised with regard to HBV-host genome integration, particularly about whether there are specific genomic regions (generally referred as hotspot sites) in the human genome that are favored for HBV integration and lead to HCC tumorigenesis and development.

To explore potential answers to these questions, we profiled 50 Chinese HCC samples by whole transcriptome sequencing (RNASeq) and array comparative genomic hybridization (aCGH), and thoroughly analyzed the HBV-human genome integration.

## Results

### Chinese HCC is predominantly associated with HBV genotype C infection

Fifty surgically resected HCC tissues from Chinese patients and 5 matched tumor adjacent normal samples were subjected to RNAseq, and their clinical information was also collected and analyzed. The transcriptome sequencing data showed that 44 HCC patients (88%, 44/50) expressed HBV encoding genes, confirming a high frequency of HBV infection in Chinese HCC patients. 77% HCC patients (34/44) were infected with genotype C HBV, and 23% (10/44) were infected with genotype B HBV (Table 1). For the 5 adjacent normal samples, 3 were infected with genotype C HBV, 1 was infected with genotype B HBV and 1 was HBV negative, which is consistent with HBV infection status in corresponding tumor samples. Furthermore, serum AFP was postive (the cutoff level of normal serum AFP is ≤20ug/L) [19]) in 72% of HCC patients with the C variant infection, compared to that in 33% of patients with the B variant infection, indicating the elevated AFP in HBV C-type patients (p = 0.052, OR = 4.99) (Table 1). No significant correlations were observed between HBV genotypes and other clinical parameters, such as age, gender, vascular invasion, tumor grade, tumor stage, tumor size, and tumor number (Table 1).

**Table 1:** Clinical Characteristics of HCC Samples.

	Genotype C (N = 34)	Genotype B (N = 10)	Negative (N = 6)	P-value
**Tumor grade**				0.78
1	1	0	1	
2	13	5	0	
3	20	5	5	
**Stage**				0.31
1	18	5	5	
2	6	4	0	
3	9	1	1	
**Gender**				0.57
M	29	10	4	
F	5	0	2	
**HBV infection type** ^**#**^				0.36
HBsAg+, HBeAb+	20	6	2	
HBsAg+, HBeAb+, HBcAb+	4	3	0	
**Tumor size** ^**#**^				0.21
Large(> = 4cm)	23	5	1	
Small(<4cm)	6	4	2	
**Cirrhosis**				0.57
Positive	30	8	3	
Negative	3	2	3	
**Tumor number** ^**#**^				0.63
Multiple	5	2	0	
Single	24	6	3	
**Vascular invasion**				0.70
Positive	10	2	1	
Negative	23	8	5	
**AFP** ^**#**^				0.05*
Positive(>20ug/L)	21	3	1	
Negative(< = 20ug/L)	8	6	2	
**HBV-MLL4 integration**				0.66
Positive	7	1		
Negative	27	9		
**Age**				0.29
≥51	16	7	3	
<51	17	3	3	
Median	49	56	54	

Note:

^#^ Some patients’ clinic information is not complete, like HBV infection type, tumor size, tumor number, AFP level.

*P-value is significant. All P-values were calculated by fisher exact test between genotype C and B group.

**Fig 1:**
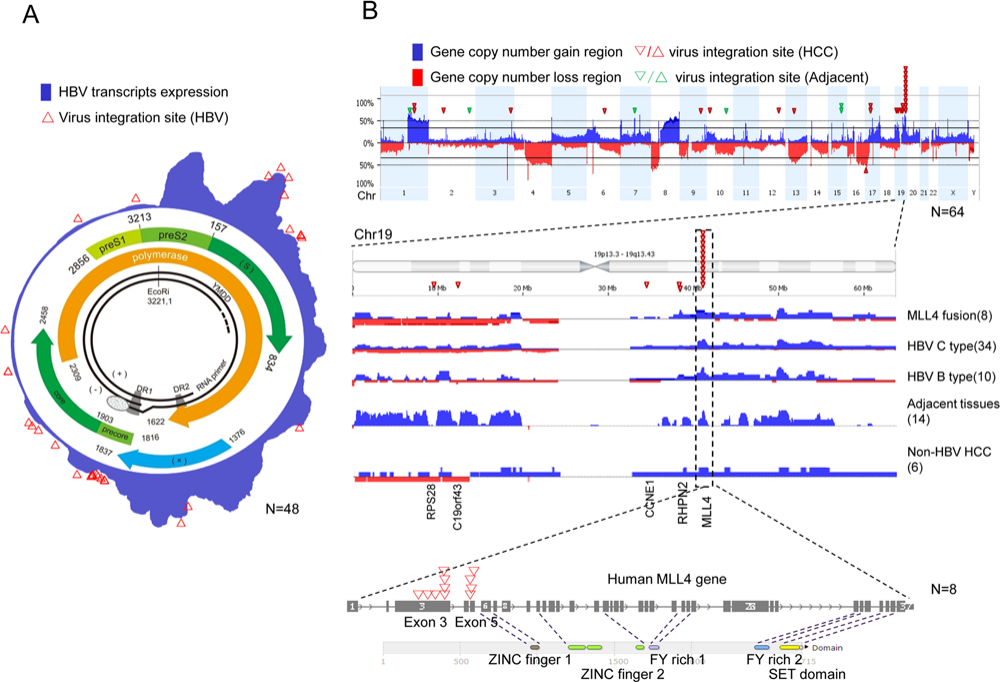
Genome landscape of HCC and related HBV. RNASeq and aCGH data in HCC tissues were analyzed. (A) Model of the HBV genotype C genome, including the well-known HBV genome within the circle, and HBV mRNA expression [blue area] and integration sites [red triangle]. (B) HCC genomic landscape by HBV integration. The gene copy number gain is shown in blue, the copy number loss in red, and the human genome sites involved in virus integration at each chromosome as a red triangle.

As shown in Fig 1A, an aggregated overview of HBV transcript expressions was generated (indicated by blue shadows) from RNASeq analysis in 44 HBV-positive tumor samples and 4 HBV-positive tumor-adjacent samples. HBV transcripts revealed a “segmented” expression pattern of the HBV genome; four expression peaks were identified in the HBx region, prior to HBx, Pre-S2, and S regions [14].

### RNASeq identified HBV-human integration transcripts

RNASeq data was quality assured with average 147M raw reads and average 80% mapping rate for each sample. Detailed QC report was shown in S1 Table. 16 of the 44 HBV-positive (36%) HCC tissues harbored HBV-human gene integration transcripts; nearly all of them (15/16) were HBV genotype C-positive, while only one case (sample 353T) was related to HBV genotype B infection. In addition, the HBV-human integration was detected in both adjacent normal tissues (green triangle) and tumor tissues (red triangle) (Fig 1B, details in S2 Table). There were 33 integration junction sites (transcripts) detected by RNASeq in the 16 samples (S2 Table). HBV S, X, and core protein ORFs were most frequently involved in the HBV-human integration transcripts, which were detected in 12, 12, and 8 integration sites, respectively. Furthermore, the DR1 region (close to the basal core promoter) and the S protein region (close to the preS2 promoter) were the two dominant integration hotspots in the HBV genome, which were involved in 55% (18/33) and 30% (10/33) integration sites identified in this cohort, respectively (Fig 1A). The integration hotspots in the human genome were observed mostly in chr19, in which there were 1, 1, 1, 2, and 11 integration transcripts (or integration) found in *PRS28*, *C19orf43*, *CCNE1*, *RHPN2*, and *MLL4*, respectively.

We then focused on further characterizing *MLL4* (mixed-lineage leukemia 4), which was the most frequent HBV integration sites (piled triangles in Fig 1B) in this cohort of patients; 18% (8/44) of the HCC patients harbored the HBV-MLL4 gene integration. A total of 11 HBV junction sites in *MLL4* were detected in eight samples, of which two junction sites were detected in three samples (S2 Table). All 11 junction sites were confirmed by PCR and Sanger sequencing (S1 Fig). Interestingly, the junctions of all the integration occurred in exon 3 and the boundary of exon 5-intron 5 of *MLL4*, right before the sequences encoding for zinc fingers DNA binding and methy-transferase SET domains (Fig 1B, bottom). These HBV integration sites in *MLL4* are consistent with previous reports [20].

We also observed that HBV integration was positively associated with gene copy number gains in chromosomes, such as chr1q, chr17 and chr19, in HCC patients (Fig 1B, up blue bars). We further analyzed the *MLL4* copy number data in 44 HBV positive tumor tissues and 14 HBV positive adjacent normal tissues and found 50/58 tissues displayed *MLL4* gene copy number gains (Fig 1B). Conversely, in 6 HBV negative tumor samples, only 3/6 samples are *MLL4* copy number increased. *MLL4* gene copy numbers tend to be increased more frequently in the HBV-positive samples than that in the HBV-negative samples (fisher-test, p-value = 0.058, OR = 6, S3 Table).

### *MLL4* is over-expressed in HCC patients with HBV-MLL4 integration

**Fig 2:**
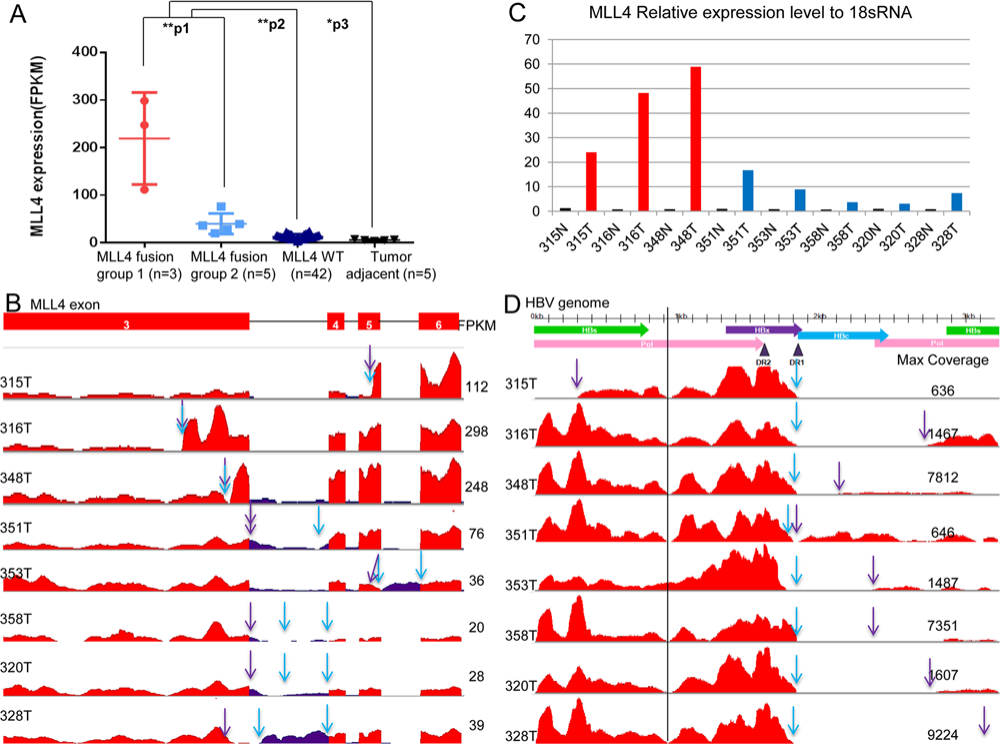
HBV transcripts and *MLL4* mRNA expression. (A) *MLL4* mRNA expression (FPKM value) comparison across four groups: the *MLL4* fusion group 1 represents HCC samples carrying HBV integration into *MLL4* exon 3 or 5; the *MLL4* fusion group 2 represents HCC samples with the HBV integration into *MLL4* introns or intron/exon boundaries; the *MLL4* WT group represent HCC samples with *MLL4* integration wild-type; and the tumor-adjacent represents HCC-adjacent normal samples. The *p*-values were determined by an unpaired one-tailed t-test, **p1 = 0.003, **p2 = 1.57E-7, *p3 = 0.03. (B) *MLL4* exon 3 to exon 6 coverage by RNA sequencing. HBV-MLL4 integration upstream junction sites are shown with purple arrows and downstream junction sites with blue arrows. The *MLL4* gene expression level was quantified as the FPKM value for each sample, as shown on the right.

*MLL4* was found to be over-expressed in HBV-MLL4 integration-positive tissues compared to the wild-type *MLL4* tissues (in both HCC and matched adjacent normal tissues) (*p*<0.01). Average mRNA levels of the 8 *MLL4*-integration-positive samples were 8.6 times higher than the *MLL4* wild-type tumors and 19.5 times higher than the adjacent tissues. Among the 8 *MLL4*-integration-positive samples, we observed two subgroups; three of them (315T, 316T, and 348T) had much higher levels of the expression than the remaining five (351T, 353T, 358T, 320T, and 328T) (*p*<0.01, Fold change = 5.5) (Fig 2A). The coverage of *MLL4* transcripts and HBV transcripts measured by RNASeq is also shown in Fig 2B and Fig 2D, respectively. We further performed real-time RT-PCR to quantify MLL4 expression in all the 8 HBV-MLL4 integration HCC samples and their adjacent normal samples. MLL4 expression level relative to 18s was shown in Fig 2C. MLL4 expression is highest in sample 315T, 316T and 348T and lowest in the 8 paired adjacent samples (0.72~1.25 fold compared to 18s). MLL4 expression levels were significantly higher in tumors compared with adjacent samples (p = 0.015), which is very consistent with RNASeq MLL4 expression data. These data indicated that HBV integration drives *MLL4* expression in HCC patients.

### Genomic DNA structures of the HBV-MLL4 integration

**Fig 3:**
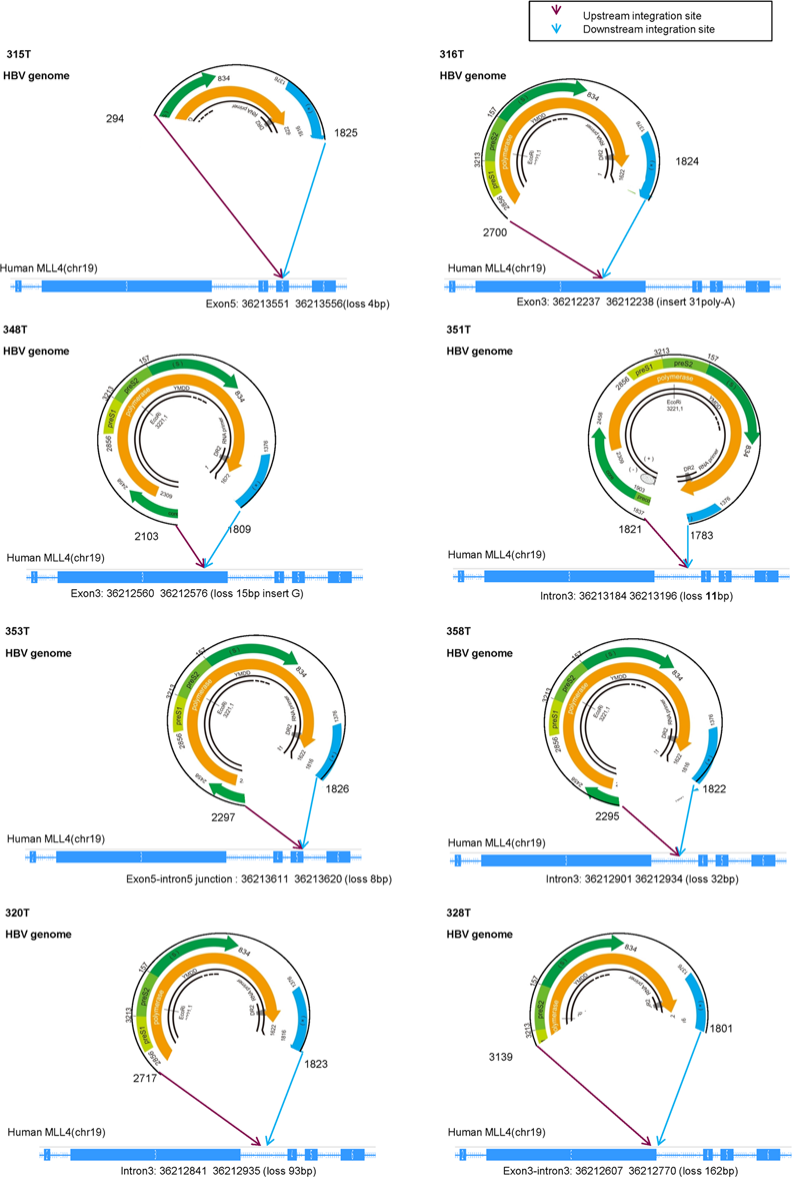
Genomic structures of HBV integration. HBV insertions are shown as circles marked with insertion positions. *MLL4* insertion sites are marked as purple (upstream) and blue arrows (downstream), noted with the exact integration position on human gene *MLL4*.

To understand the potential mechanism for the *MLL4* mRNA over-expression, we used long-range PCR method with primers derived from *MLL4* exons 3 and 6 to comprehensively characterize the genomic structures of the integration genes. As shown in Fig 3, in patient 315T, a segment of the HBV genome-minus strand, ranging from 294bp to 1825bp, was inserted into human *MLL4* exon 5. At the same time, a 4bp deletion occurred in exon 5. The integration resulted in a direct integration of HBV HBx open reading frame with the human *MLL4* gene (Fig 3). Similar observations were found in 7 other cases, in which HBV minus chain was inserted into one of human *MLL4* alleles, where there were 4~162bp deletions in *MLL4*, with an additional poly-A insertion in patient 316T. For HBV, although the upstream junction sites varied from HBx, HBc to HBs, the downstream junction sites all occurred in HBx, near the DR2 to DR1 region, consistent with the integration hotspots reported [21].

Consistent with the genomic structures of the HBV-MLL4 integration, we found that only the inserted genomic fragments of HBV were expressed and detected by RNASeq (Fig 2D). For example, HBV transcripts expression was detected only for the region from 294nt to 1825nt in patient sample 315T. The expressed region of HBV transcripts perfectly matched the inserted genomic sequences (Fig 3). Similar observations were found in 7 other samples. It is worth noting that there was almost no coverage around the 930bp position after HBs, suggesting that the HBs transcripts stopped here and its downstream HBx-MLL4 formed new transcripts (Fig 2D, vertical line). For the first time, we captured the comprehensive genomic insertion sequence for HBV, which determines the HBV transcripts’ expression in humans.

### HBV integration altered *MLL4* transcription via alternative splicing machinery

**Fig 4:**
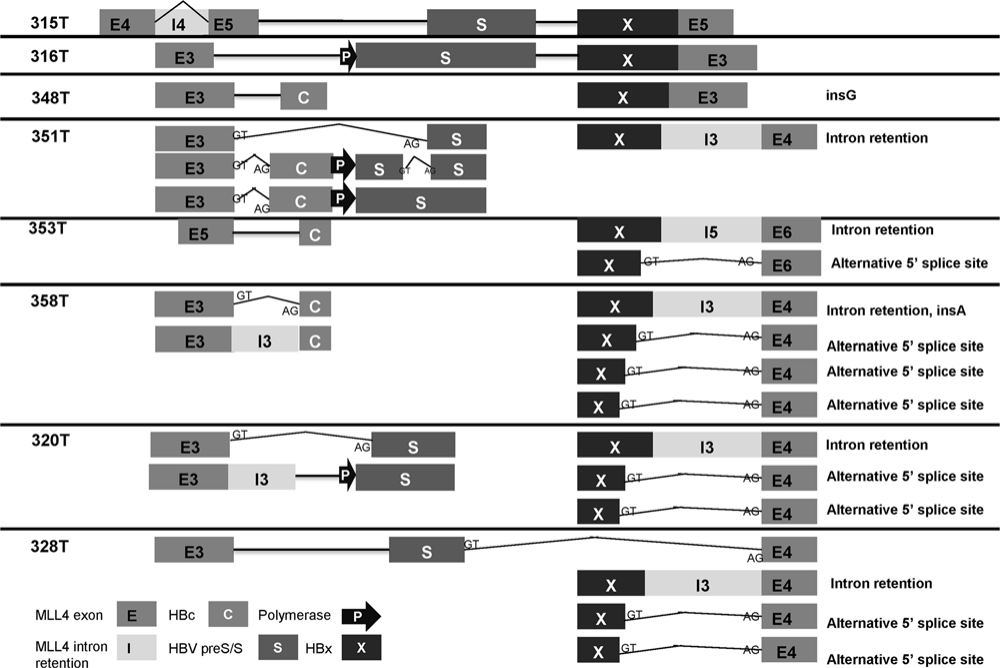
cDNA structure of HBV integration. Cartoon illustrating HBV-integrated cDNA structures. Full-length (315T, 316T, and 328T) and partially inserted cDNA sequences are shown for each sample, noted with alternative splicing isoforms for each transcript.

When MLL4-HBV integration junctions were all confirmed at cDNA levels (S1 Fig), we found that in patients 315T, 316T, 328T, and 353T, the junctions were also detected in adjacent normal tissues (S2 Fig). In 328T and 353T, the PCR bands were much weaker in the adjacent normal tissues. There are two possible explanations: one could be a mixture of some tumor cells in adjacent normal tissues; the other could be that HBV integration is a pre-cancerous event occurring in normal cells. Moreover, multiple PCR products were found in most of the junction sites (the numbers of main PCR products are shown in S2 Fig). Sequencing analysis of those PCR products revealed that in 7 out of the 11 junction sites, there was more than one cDNA structure (S4 Table). As shown in Fig 4, sequences of the shorter transcripts were the same as the RNASeq sequences, whereas the longer transcripts consist of *MLL4* sequences of gDNA structure including introns. Furthermore, HBV sequence deletions were found in two additional transcripts in 351T and 353T (the 351T-2 upstream junction and the 353T downstream junction, S4 Table).Using a similar long-range PCR method with paired primers from *MLL4* exons 3 and 6, we further characterized the HBV integration structures of the transcripts (S2 Fig). In 315T and 316T, Sanger sequencing confirmed that the cDNA sequence was exactly the same as the genomic DNA sequence for HBV-*MLL4* integration. In 328T, the up-stream junction from *MLL4* to HBV was matched between the genomic DNA and cDNA; in the downstream junction, *MLL4* was spliced to exon 4 with a loss of 162bp in genomic DNA (S2 Fig). On the other hand, partial HBs and the entire HBx of HBV were also lost during transcription. We did not find HBV-inserted PCR products in the other 5 samples, 351T, 348T, 353T, 358T, and 320T, under these experimental conditions.

Hence, we hypothesize that the HBx promoter potentially initiated a new transcript starting from the HBV genome. To illustrate this, we used primer from *HBx* to *MLL4* exon 6 to capture downstream structures of the integration. We found multiple PCR products, ranging from 1 to 4, in each of the 8 samples (S2 Fig), with several interesting observations (shown in Fig 4 and S5 Table):

First, when downstream integration occurred in *MLL4* exons (315T, 316T, 348T), there was only one transcript detected in each sample, as there was no alternative splicing at the integration junction site. In contrast, when downstream integration occurred in *MLL4* introns or exon-intron boundaries (353T, 358T, 320T, and 328T), there were 2 to 4 transcripts detected in each sample; there was one exception observed in 351T, which had only one downstream transcript as a result of intron retention, despite the fact that there were three transcripts in the upstream junction.

Second, for those samples with more than one downstream transcript, the major transcripts (brighter PCR bands shown in S2 Fig) had no splicing, whereas the minor transcripts skipped *MLL4* introns and spliced to the next exon. In samples 320T, 328T, and 358T, although their DNA integration sites were different, we found two 5’ donor sites (GT) in the HBV insertion sequence at HBx 1634 nt and 1646 nt, respectively.

Third, when we looked further into the *MLL4* mRNA expression patterns of each of the three samples 315T, 316T, and 348T in the “high” subgroup, in which HBV was inserted to *MLL4* exons, it clearly showed that *MLL4* expression was dramatically elevated for the exons right after the integration junction sites, as indicated by the blue and the purple arrows in Fig 2B. In contrast, there were no obvious differences in *MLL4* expression regardless of the upstream junction sites and/or downstream integration junction sites in samples 351T, 358T, 320T, 328T, and 353T in the “low” subgroup, in which multiple transcripts were detected (S2 Fig). In 328T, although the *MLL4*-*HBV* upstream junction site was in exon 3, the expression of *MLL4* exon 3 was decreased right after the integration site, which is consistent with its genomic sequence, in that exon 3 was partially lost due to the HBV integration (Figs 3 and 4).

### *HBV-MLL4* integration is associated with a distinct HCC patient segment with differential gene expression

We also assessed the relationship of the *MLL4* integration with the well-characterized genetic alternations in HCC, including *CTNNB1* and *TP53* mutations. As shown in Table 2, there was one *MLL4*-integration-positive patient (1/8; 12.5%) harboring a *CTNNB1* mutation and an additional two (2/8; 25%) patients having *TP53* mutations. The frequency was lower than that of the non-integration patients, of whom 19.0% (8/42) had *CTNNB1* mutations (p>0.05, OR = 0.61) and 45.2% (19/42) had *TP53* mutations (p>0.05, OR = 0.40). In addition, the *MLL4* integration was mutually exclusive with *c-Met* gene amplification in this cohort of patients. In summary, these data suggest that *HBV-MLL4* integration may represent a unique molecular segment of HCC.

**Table 2:** Major genetic changes in HCC samples.

	HBV transcripts positive																																												HBV negative					
	HBV-MLL4+								HBV-MLL4 negative																																									
Sample	315T	316T	348T	351T	358T	320T	328T	353T	322T	363T	362T	332T	317T	329T	307T	312T	330T	323T	360T	335T	346T	354T	361T	334T	345T	304T	342T	350T	310T	352T	311T	349T	355T	314T	325T	305T	319T	339T	326T	327T	347T	321T	333T	309T	308T	337T	336T	356T	359T	357T
HBV-MLL4	◆	◆	◆	◇	◇	◇	◇	◇																																										
TP53	×	*							*	*	*	*	*	×	×	×	×	×	×	×	×	×	×	×								×													×	×				
CTNNB1							*													*					*	*	*	*	*																		*	*		
c-Met																				○										○																				

^◆^ HBV-MLL4 integration in exon;

^◇^ HBV-MLL4 integration in intron;

* hotspot mutations, including TP53 R249S and CTNNB1 mutations;

^×^ TP53 non-hotspot mutations;

^○^ c-Met gene amplificat

**Fig 5:**
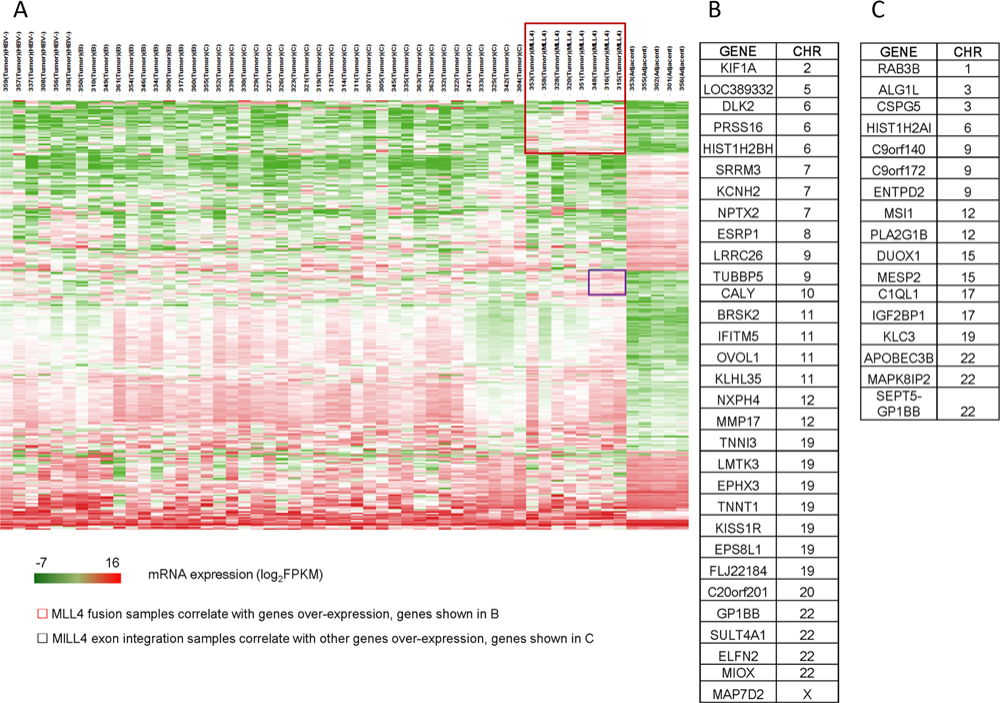
Gene expression profile of HCC harboring MLL4 integration. Unsupervised hierarchical clustering of mRNA expression across all 50 HCC samples with 4 adjacent normal samples. (A) Heatmap overview of the gene expression pattern. (B) A list of 31 genes that were distinctly over-expressed in the 8 *MLL4* integration positive HCC samples. (C) An additional 17 genes that were distinctly over-expressed in 3 higher-expressed *MLL4* HCC samples carrying *MLL4* exon integration.

*MLL4* codes for a histone methytransferase. We therefore analyzed the gene expression profiles of the HCC samples harboring *MLL4*-integration verses the wild-type, and the expression profiles of the “high” *MLL4* expression subgroup verses the “low” subgroup within the MLL4 integration-positive HCC samples. As shown in Fig 5, we observed a distinct gene expression pattern in patients harboring *MLL4* integration/over-expression by analyzing the unsupervised clustering of HCC genome expression data; a total of 31 genes were distinctly over expressed in the *MLL4* integration samples, including *LMTK3* and *KISS1R*, which were reported to be involved in oncogenesis. Moreover, 17 additional genes exhibited elevated expression distinctly in the *MLL4* expression “high” subgroup compared to the “low” sub-group.

## Discussion

HBV infection is one of important ideologies in HCC development in China [1]. Based on the analysis of a cohort of 50 Chinese HCC patients, using RNASeq and aCGH profiling, we report a number of genomic alternations as a consequence of HBV infection. Our objective was to gain molecular insight of HBV integration that may contribute to HCC tumorigenesis. In this study we identified 33 HBV-host integration transcripts in 36% (16/44) of HBV-positive HCC patients. In line with recent publications, HBV integrations to MLL4, FN1, and CCNE1 were also found in this cohort of patients. It is worth noting that HBV-MLL4 integration was greatly enriched in this cohort of HCC patients, accounting for 18% (8/44) of the patients. Sung et al reported 18 TERT integration in 81 HBV positive (unknown subtype) HCC patients from Queen Mary Hospital in Hong Kong [20]. However, we did not find any HBV-TERT integration in our cohort of 44 HBV positive samples (major HBV C type) from Zhongshan Hospital in Shanghai. We think HBV-TERT integration frequency might be different in different population and HBV subtype. Also due to the relative small sample size in this cohort, the new and missed discoveries might need to be confirmed in a larger cohort.

HBV-MLL4 integration was first reported in HCC by Saigo et al [22], in which HBV was integrated into *MLL4* intron 3. Recently, *MLL4* exons 3 and 6 were also identified as integration locations for HBV in addition to intron 3 [20]. Besides the reported integration regions, our analysis is the first to reveal that exon 5, exon 3-intron 3, and exon 5-intron 5 of *MLL4* were also frequently targeted by HBV integration in HCC patients (Fig 3). We observed insertions or deletions for both the HBV genome and the human genome during integration. More significantly, we are the first to comprehensively characterize both gDNA and cDNA sequencing and structures of these 8 recurrent HBV-MLL4 integration(Figs 3 and 4; S5 Table), which provided molecular regulation of the HBV-MLL4 transcripts. This finding made *MLL4* the most prominent gene targeted by HBV integration in HCC. HBV insertion into *MLL4* exon resulted in much higher expression of MLL4 mRNA; the exons immediately following the integration sites were extremely highly expressed, which is probably driven by the *HBx* promoter. More interestingly, the integration into *MLL4* introns led to a different expression pattern; there was no striking over-expression of *MLL4* exons immediately following the integration sites. Instead, abnormal intron expression was detected (Fig 2B). This observation suggests that *HBx* promoter efficiency might be different when inserted into different genomic locations. The *HBx* promoter would have much higher efficiency in exon regions in driving gene transcription. Another potential explanation is the interaction between the spliceosome complex and gene transcription [23]. Intron insertion might disrupt *MLL4* splicing patterns, which results in lower transcription efficiency. This splicing abnormality was further supported by the observation of multiple *MLL4* splicing variants detected in the intron-integrated cases (Fig 4); the major transcripts kept introns without splicing them, while the minor transcripts skipped introns by splicing to their next exon.

Sequencing analysis of the HBx-MLL4 integration transcripts predicted that some of the in-frame integration proteins would be translated, while other out-of-frame ones would not. For the samples, there was only one HBx-MLL4 transcript in sample 315T that was predicted to be in frame, while the HBx-MLL4 transcripts in samples 316T, 348T, and 351T were predicted to be out of frame. There might be one in-frame *HBc-MLL4* integration protein in sample 316T. In the rest of the four remaining samples with multiple transcripts, all of the transcripts spliced to exons are predicted to be in frame, while the rest meet stop codons in intron regions (S5 Table). Antibodies specifically recognizing each of these individual integration proteins would need to be generated not only to confirm the prediction, but also to be a useful tool to facilitate further biological function validation of these *MLL4* integration-.

Compared to other solid malignancies, the development of targeted therapies against HCC has been lagging, largely due to the lack of identified oncogenic drivers. The *TP53* and *CTNNB1* hotspot mutations and their functions in HCC development have been well studied previously [1]. Therapeutics against *TP53* and *CTNNB1* mutations are now in early clinical development [24,25]. However, for those patients who are lacking *TP53* and *CTNNB1* mutations, novel therapeutic targets are needed. Frequent HBV-MLL4 integration in HCC patients could provide a potential targeted therapy opportunity. *MLL4* was first reported to be amplified in 2 of 14 pancreatic carcinoma cell lines and 1 of 5 glioblastoma cell lines [26]. *MLL4* inactivation led to embryonic lethality in mice, indicating that *MLL4* is not functionally redundant with other structurally homologous *MLL* family members [27]. *MLL4* was also reported to regulate cell-cycle progression and cell viability, and the depletion of *MLL4* suppresses the growth of xenografted tumour *in vivo* [28]. Apparently, pre-clinical efforts to validate the function of the *MLL4* integration and its role in HCC tumorigenesis are warranted before developing a therapeutic modality.

In summary, we observed high-frequency HBV-MLL4 integration (18%) in Chinese HBV-positive HCC patients. *MLL4* over-expression was further driven by the *HBx* promoter and regulated by alternative splicing. It may represent a unique disease segment in HBV-positive HCC patients and a potential therapeutically opportunity for HCC patients.

## Materials and Methods

### Patients and specimens

HCC specimens were acquired from 50 patients who underwent surgical resection in Zhongshan Hospital (Shanghai, China). The characteristics of the patients are listed in Table 1. 50 Fresh HCC specimens and 5 matched adjacent normal liver tissues were used for RNA sequencing. The same 50 tumor samples and 14 matched adjacent normal liver tissues were subjected for aCGH profiling.

### Ethics Statement

Prior written informed consent was obtained from each patient, and the study was conducted in accordance with the principles of the Declaration of Helsinki and approved by the institutional review board (IRB). The name of the IRB is Biomedical Research Ethics Committee, Zhongshan Hospital, Fudan University and the project name is genetic characteristics and molecular segmentation study of hepatocellular carcinoma and the approval number is NO.2009-090.

### RNASeq and aCGH profiling

Total RNA and genomic DNA were extracted from the snap-frozen HCC or adjacent normal liver tissues, as we previously reported [29]. RNA was sequenced using an Illumina HiSeq2000 Genome Analyzer and DNA was profiled with an Agilent 244K array CGH platform according to the manufacturers’ specifications, respectively.

### Data Analysis

Gene copy number alternations were measured by log ratios using BioDiscovery Nexus Copy Number software (http://www.biodiscovery.com/software/nexus-copy-number/). An ArrayCGH log_2_ratio greater than 0.11 was considered as gene copy number gain.

Gene expression calling, integration transcript calling, and mutation calling were performed with the commercial software OmicSoft (http://www.omicsoft.com/). HBV and human gene expression quantification was measured using the FPKM value, which stands for “sequenced fragments per kilobase of exon per million fragments mapped to the human genome”.

$$\text{FPKM=}\frac{10^{9}*N}{L*R}$$

N: number of reads mapped in a gene

L: gene length (bp) (introns excluded)

R: number of raw reads

MET amplification is determined by aCGH data with log2ratio greater than 0.8, CTNNB1 and TP53 mutations were called from the RNAseq data. Allele frequencies below 10% were removed. The CTNNB1 and TP53 mutations were further confirmed by the PCR and Sanger sequencing.

Eight distinct HBV genotypes (A–H) were downloaded from NCBI and built together as the HBV reference genome. We mapped the RNASeq raw reads for each sample to the HBV genome. If any reads were uniquely mapped to either subtype, we classified the sample to that subtype. Then, we combined Human Genome Build 37 and HBV genotypes B and C together as a reference to detect the human gene-HBV integration. We improved the parameters in the ArrayStudio semap module for integration detection [30].

The aCGH and RNASeq profiling data have been deposited to the GEO database (Gene Expression Omnibus) under accession number GSE65484 and GSE65485. Superseries accession GSE65486 will link the CGH and RNA-Seq studies together.

### *MLL4* expression quantification by real-time RT-PCR

RNA was reversely transcribed into cDNA using a PrimeScript RT reagent kit with a gDNA Eraser (Perfect Real Time) (TaKaRa Biotechnology, Dalian, China) according to the manufacturer’s instructions. Reactions of real-time PCR were performed on ABI 7900 HT Sequence Detection System (Applied Biosystems) using TaqMan Gene Expression Master Mix (4369016, Applied Biosystems) according to the manufacturer’s instructions. TaqMan Gene Expression Assay (Hs00207065_m1, Applied Biosystems) was used to detect MLL4 expression level. MLL4 values are shown as relative expression levels to 18s (4319413, Applied Biosystems) using formula 2^-ΔCt (MLL4-18s)^*10^5^.

### HBV integration junction site confirmation

PCR was performed on cDNA using an AmpliTaq Gold^360^ Master Mix (Life Technologies) according to the manufacturer’s instructions. The PCR products of both tumor tissues and paired normal tissues were characterized by their correct size using agarose gel electrophoresis. The PCR products of tumor tissues were sequenced through Sanger sequencing (S4 Table).

### HBV insertion sequence identification

Genomic DNAs and cDNAs from tumor tissues were amplified by PCR using Premix LA Taq DNA Polymerase (TaKaRa Biotechnology, Dalian, China) according to the manual’s instructions. Long range PCR Primer sequences used for determining gDNA and cDNA of HBV-MLL4 integration was shown in S6 Table. PCR products were purified with an Agarose Gel DNA Extraction Kit (magnetic beads purification) (Shanghai Somic Biological Technology, Shanghai, China) and further sequenced by Sanger sequencing.

## Supporting Information

S1 Fig Sequence confirmation of HBV-MLL4 fusion junction sites. Sanger sequencing confirmed the 11 junction sites detected by RNASeq. For 353T downstream junction site and 358T upstream site, Sanger sequencing confirmed additional junction sites.
(TIF)

S2 Fig PCR products of HBV-MLL4 junction sites and cDNA structure. (A) PCR gel of integration junctions in the 8 tumor samples and matched adjacent normal, using GAPDH as control. (B) PCR gel figure of HBV integration cDNA structures using long-range PCR assay with primers from *MLL4* exons 3 and 6. (C) PCR gel figure of HBV integration cDNA structures using long-range PCR assay with primers from *HBx* to *MLL4* exon 6.
(TIF)

S1 Table RNASeq data QC report. (DOCX)

S2 Table All HBV-human fusions detected. (DOCX)

S3 Table MLL4 gene copy number gain in HBV positive samples. (DOCX)

S4 Table cDNA sequences of HBV-MLL4 junction sites .
(DOCX)

S5 Table gDNA and cDNA structure of HBV-MLL4 integration. (DOCX)

S6 Table Primer sequences used for determining HBV-MLL4 integration. (DOCX)

## References

[1] El-Serag HB. Hepatocellular carcinoma. N Engl J Med. 2011;365(12):1118–27. 10.1056/NEJMra1001683 21992124

[2] Szymanska K, Chen JG, Cui Y, Gong YY, Turner PC, Villar S, et al TP53 R249S mutations, exposure to aflatoxin, and occurrence of hepatocellular carcinoma in a cohort of chronic hepatitis B virus carriers from Qidong, China. Cancer Epidemiol Biomarkers Prev. 2009;18(5):1638–43. 10.1158/1055-9965.EPI-08-1102 19366907

[3] Guichard C, Amaddeo G, Imbeaud S, Ladeiro Y, Pelletier L, Maad IB, et al Integrated analysis of somatic mutations and focal copy-number changes identifies key genes and pathways in hepatocellular carcinoma. Nat Genet. 2012;44(6):694–8. 10.1038/ng.2256 22561517 PMC3819251

[4] McGlynn KA, London WT. The global epidemiology of hepatocellular carcinoma: present and future. Clin Liver Dis. 2011;15(2):223–43, vii–x. 10.1016/j.cld.2011.03.006 21689610 PMC4141529

[5] Welzel TM, Graubard BI, Zeuzem S, El-Serag HB, Davila JA, McGlynn KA. Metabolic syndrome increases the risk of primary liver cancer in the United States: a study in the SEER-Medicare database. Hepatology. 2011;54(2):463–71. 10.1002/hep.24397 21538440 PMC4141525

[6] Sherman M. Hepatocellular carcinoma: epidemiology, surveillance, and diagnosis. Semin Liver Dis. 2010;30(1):3–16. 10.1055/s-0030-1247128 20175029

[7] Paraskevis D, Magiorkinis G, Magiorkinis E, Ho SY, Belshaw R, Allain JP, et al Dating the origin and dispersal of hepatitis B virus infection in humans and primates. Hepatology. 2013;57(3):908–16. 10.1002/hep.26079 22987324

[8] KRaC WH. Hepatitis B virus. London, UK: Imperial College Press; 1998.

[9] Merican I, Guan R, Amarapuka D, Alexander MJ, Chutaputti A, Chien RN, et al Chronic hepatitis B virus infection in Asian countries. J Gastroenterol Hepatol. 2000;15(12):1356–61. 11197043 10.1046/j.1440-1746.2000.0150121356.x

[10] Chu CM. Natural history of chronic hepatitis B virus infection in adults with emphasis on the occurrence of cirrhosis and hepatocellular carcinoma. J Gastroenterol Hepatol. 2000;15 Suppl:E25–30. 10921378 10.1046/j.1440-1746.2000.02097.x

[11] Lu T, Seto WK, Zhu RX, Lai CL, Yuen MF. Prevention of hepatocellular carcinoma in chronic viral hepatitis B and C infection. World J Gastroenterol. 2013;19(47):8887–94. 10.3748/wjg.v19.i47.8887 24379612 PMC3870540

[12] Aspinall EJ, Hawkins G, Fraser A, Hutchinson SJ, Goldberg D. Hepatitis B prevention, diagnosis, treatment and care: a review. Occup Med (Lond). 2011;61(8):531–40. 10.1093/occmed/kqr136 22114089

[13] Kim CM, Koike K, Saito I, Miyamura T, Jay G. HBx gene of hepatitis B virus induces liver cancer in transgenic mice. Nature. 1991;351(6324):317–20. 2034275 10.1038/351317a0

[14] Moolla N, Kew M, Arbuthnot P. Regulatory elements of hepatitis B virus transcription. J Viral Hepat. 2002;9(5):323–31. 12225325 10.1046/j.1365-2893.2002.00381.x

[15] Kremsdorf D, Soussan P, Paterlini-Brechot P, Brechot C. Hepatitis B virus-related hepatocellular carcinoma: paradigms for viral-related human carcinogenesis. Oncogene. 2006;25(27):3823–33. 16799624 10.1038/sj.onc.1209559

[16] Huang J, Deng Q, Wang Q, Li KY, Dai JH, Li N, et al Exome sequencing of hepatitis B virus-associated hepatocellular carcinoma. Nat Genet. 2012;44(10):1117–21. 10.1038/ng.2391 22922871

[17] Toh ST, Jin Y, Liu L, Wang J, Babrzadeh F, Gharizadeh B, et al Deep sequencing of the hepatitis B virus in hepatocellular carcinoma patients reveals enriched integration events, structural alterations and sequence variations. Carcinogenesis. 2013;34(4):787–98. 10.1093/carcin/bgs406 23276797

[18] Lau CC, Sun T, Ching AK, He M, Li JW, Wong AM, et al Viral-human chimeric transcript predisposes risk to liver cancer development and progression. Cancer Cell. 2014;25(3):335–49. 10.1016/j.ccr.2014.01.030 24582836

[19] Farinati F, Marino D, De Giorgio M, Baldan A, Cantarini M, Cursaro C, et al Diagnostic and prognostic role of alpha-fetoprotein in hepatocellular carcinoma: both or neither? Am J Gastroenterol. 2006;101(3):524–32. 16542289 10.1111/j.1572-0241.2006.00443.x

[20] Sung WK, Zheng H, Li S, Chen R, Liu X, Li Y, et al Genome-wide survey of recurrent HBV integration in hepatocellular carcinoma. Nat Genet. 2012;44(7):765–9. 10.1038/ng.2295 22634754

[21] Jiang Z, Jhunjhunwala S, Liu J, Haverty PM, Kennemer MI, Guan Y, et al The effects of hepatitis B virus integration into the genomes of hepatocellular carcinoma patients. Genome Res. 2012;22(4):593–601. 10.1101/gr.133926.111 22267523 PMC3317142

[22] Saigo K, Yoshida K, Ikeda R, Sakamoto Y, Murakami Y, Urashima T, et al Integration of hepatitis B virus DNA into the myeloid/lymphoid or mixed-lineage leukemia (MLL4) gene and rearrangements of MLL4 in human hepatocellular carcinoma. Hum Mutat. 2008;29(5):703–8. 10.1002/humu.20701 18320596

[23] Ji X, Fu XD. The mediator couples transcription and splicing. Mol Cell. 2012;45(4):433–4. 10.1016/j.molcel.2012.02.003 22365824 PMC3292770

[24] Wang Z, Sun Y. Targeting p53 for Novel Anticancer Therapy. Transl Oncol. 2010;3(1):1–12. 20165689 10.1593/tlo.09250 PMC2822448

[25] Voronkov A, Krauss S. Wnt/beta-catenin signaling and small molecule inhibitors. Curr Pharm Des. 2013;19(4):634–64. 23016862 10.2174/138161213804581837 PMC3529405

[26] Huntsman DG, Chin SF, Muleris M, Batley SJ, Collins VP, Wiedemann LM, et al MLL2, the second human homolog of the Drosophila trithorax gene, maps to 19q13.1 and is amplified in solid tumor cell lines. Oncogene. 1999;18(56):7975–84. 10637508 10.1038/sj.onc.1203291

[27] Glaser S, Schaft J, Lubitz S, Vintersten K, van der Hoeven F, Tufteland KR, et al Multiple epigenetic maintenance factors implicated by the loss of Mll2 in mouse development. Development. 2006;133(8):1423–32. 16540515 10.1242/dev.02302

[28] Ansari KI, Kasiri S, Mishra BP, Mandal SS. Mixed lineage leukaemia-4 regulates cell-cycle progression and cell viability and its depletion suppresses growth of xenografted tumour in vivo. Br J Cancer. 2012;107(2):315–24. 10.1038/bjc.2012.263 22713656 PMC3394987

[29] Zhang XC, Zhang J, Li M, Huang XS, Yang XN, Zhong WZ, et al Establishment of patient-derived non-small cell lung cancer xenograft models with genetic aberrations within EGFR, KRAS and FGFR1: useful tools for preclinical studies of targeted therapies. J Transl Med. 2013;11:168 10.1186/1479-5876-11-168 23842453 PMC3716998

[30] Ge H, Liu K, Juan T, Fang F, Newman M, Hoeck W. FusionMap: detecting fusion genes from next-generation sequencing data at base-pair resolution. Bioinformatics. 2011;27(14):1922–8. 10.1093/bioinformatics/btr310 21593131

