# Supplementary figures and images for: Identification of HBV-MLL4 Integration and Its Molecular Basis in Chinese Hepatocellular Carcinoma
Source: PLoS One. 2015 Apr 22;10(4):e0123175. doi: 10.1371/journal.pone.0123175 (PMC4406717; doi:10.1371/journal.pone.0123175)

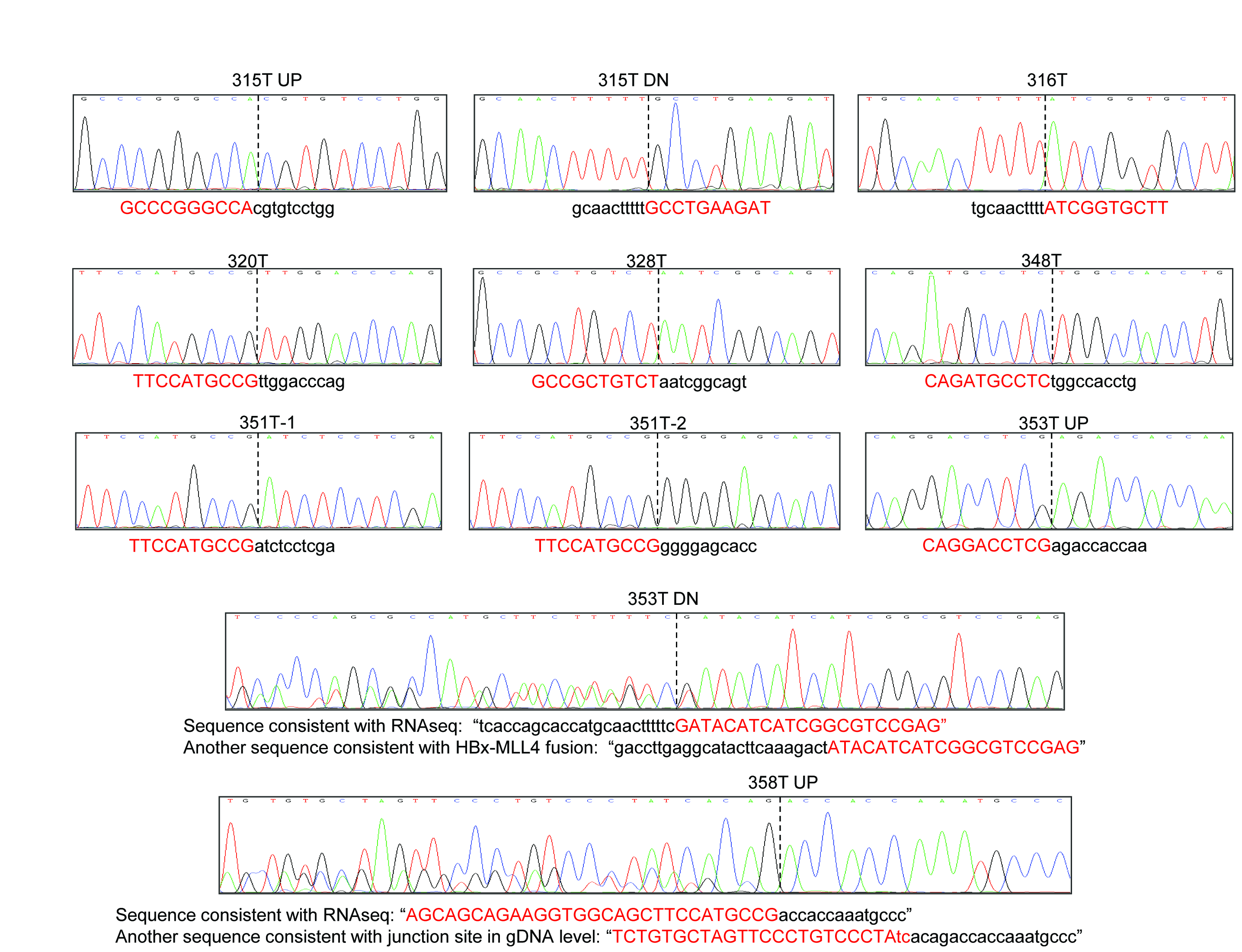

Supplement: S1 Fig — Sanger sequencing confirmed the 11 junction sites detected by RNASeq. For 353T downstream junction site and 358T upstream site, Sanger sequencing confirmed additional junction sites. (TIF) [file pone.0123175.s001.tif]

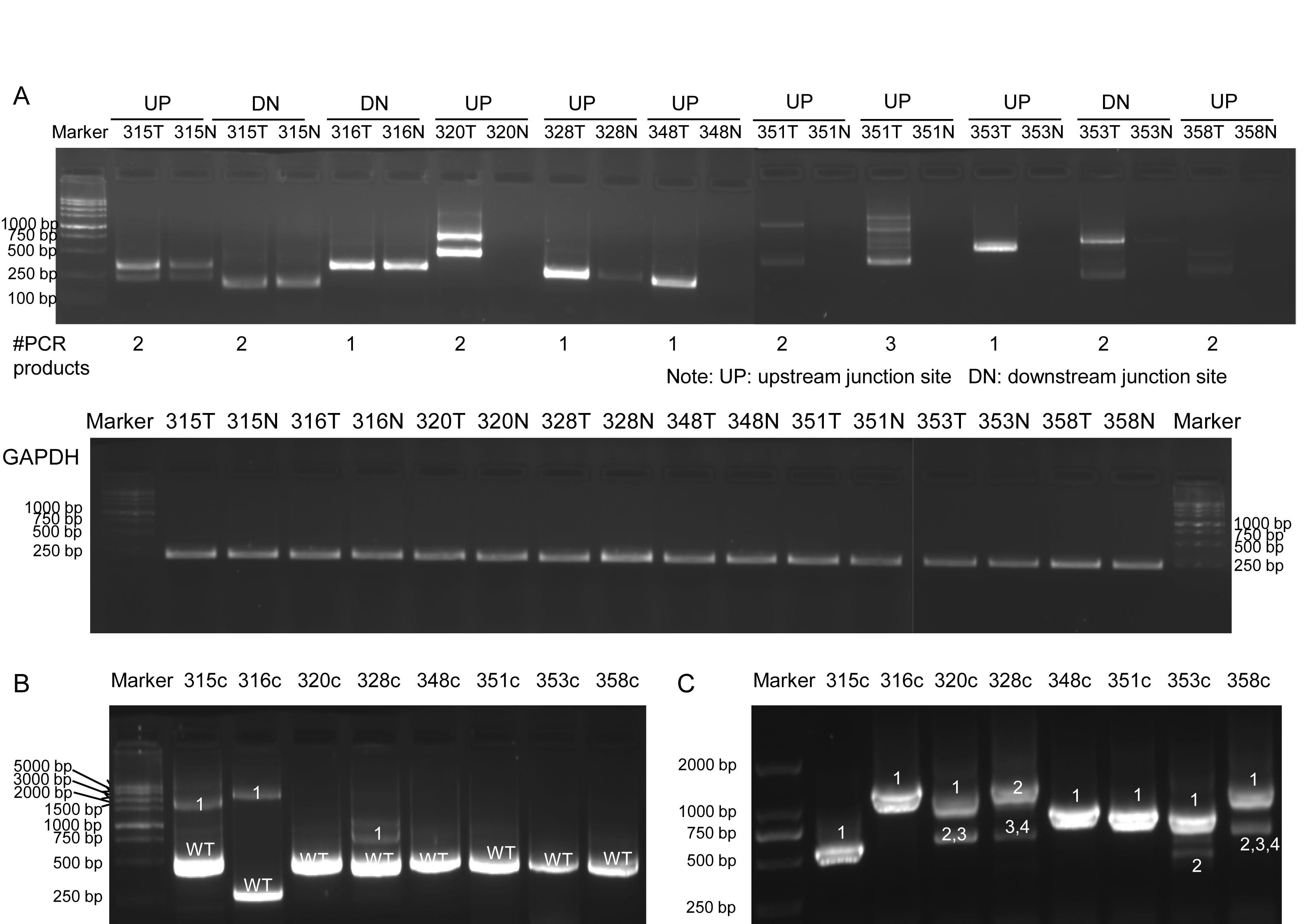

Supplement: S2 Fig — (A) PCR gel of integration junctions in the 8 tumor samples and matched adjacent normal, using GAPDH as control. (B) PCR gel figure of HBV integration cDNA structures using long-range PCR assay with primers from MLL4 exons 3 and 6. (C) PCR gel figure of HBV integration cDNA structures using long-range PCR assay with primers from HBx to MLL4 exon 6. (TIF) [file pone.0123175.s002.tif]
