# Supplementary material for: Identification of HBV-MLL4 Integration and Its Molecular Basis in Chinese Hepatocellular Carcinoma
Source: PLoS One. 2015 Apr 22;10(4):e0123175. doi: 10.1371/journal.pone.0123175 (PMC4406717; doi:10.1371/journal.pone.0123175)
Supplement: S1 Table — (DOCX) [file pone.0123175.s003.docx]

**S1 Table. RNASeq data QC report.**

| **Pair** | **Mean**  **Insert Size** | **Total read#** | **Uniquely paired read#** | **Uniquely mapped read1#** | **Uniquely mapped read2#** | **Unmapped read1#** | **Unmapped read2#** | **Total mapping rate%** |
| --- | --- | --- | --- | --- | --- | --- | --- | --- |
| 304T | 297.2 | 154,203,168 | 110,050,666 | 8,369,757 | 8,022,626 | 13,706,494 | 14,053,625 | 82.0 |
| 305T | 283.4 | 140,908,874 | 116,709,896 | 5,325,609 | 4,698,842 | 6,773,880 | 7,400,647 | 89.9 |
| 307T | 311.6 | 138,207,942 | 111,069,898 | 6,250,398 | 5,749,695 | 7,318,624 | 7,819,327 | 89.0 |
| 308T | 310.6 | 142,291,168 | 113,621,100 | 7,168,682 | 5,579,667 | 7,166,352 | 8,755,367 | 88.8 |
| 309T | 312.6 | 172,521,834 | 121,225,456 | 9,116,729 | 8,265,110 | 16,531,460 | 17,383,079 | 80.3 |
| 310T | 292.1 | 98,462,360 | 71,031,996 | 3,550,051 | 3,135,006 | 10,165,131 | 10,580,176 | 78.9 |
| 311T | 320.4 | 143,298,326 | 107,878,664 | 9,923,924 | 6,097,578 | 7,785,907 | 11,612,253 | 86.5 |
| 312T | 308.1 | 178,356,808 | 147,451,714 | 5,592,070 | 4,889,089 | 9,860,477 | 10,563,458 | 88.5 |
| 314T | 285.1 | 127,055,646 | 101,076,530 | 5,345,766 | 4,347,597 | 7,643,792 | 8,641,961 | 87.2 |
| 315T | 307.9 | 154,122,014 | 110,249,178 | 7,659,567 | 7,949,538 | 14,276,851 | 13,986,880 | 81.7 |
| 316T | 305.7 | 116,326,652 | 79,417,450 | 5,754,436 | 6,820,793 | 12,700,165 | 11,633,808 | 79.1 |
| 317T | 291.7 | 119,802,904 | 79,018,912 | 5,992,897 | 6,481,426 | 14,399,099 | 13,910,570 | 76.4 |
| 319T | 292.9 | 136,537,640 | 96,844,470 | 6,548,255 | 6,755,208 | 13,298,330 | 13,091,377 | 80.7 |
| 320T | 278.1 | 143,188,102 | 98,939,256 | 6,245,121 | 5,844,863 | 15,879,302 | 16,279,560 | 77.5 |
| 321T | 289.5 | 143,588,700 | 105,505,936 | 7,462,400 | 6,402,298 | 11,578,982 | 12,639,084 | 83.1 |
| 322T | 301.0 | 152,661,302 | 111,074,826 | 8,115,749 | 8,251,720 | 12,677,489 | 12,541,518 | 83.5 |
| 323T | 287.7 | 126,136,174 | 94,077,988 | 5,820,100 | 4,764,117 | 10,208,993 | 11,264,976 | 83.0 |
| 325T | 295.5 | 122,555,560 | 92,753,024 | 5,778,259 | 5,343,723 | 9,123,009 | 9,557,545 | 84.8 |
| 326T | 280.1 | 128,607,300 | 93,638,646 | 5,519,835 | 4,856,457 | 11,964,492 | 12,627,870 | 80.9 |
| 327T | 306.3 | 123,831,820 | 86,453,742 | 8,103,106 | 7,676,793 | 10,585,933 | 11,012,246 | 82.6 |
| 328T | 313.4 | 118,436,560 | 82,004,780 | 7,097,718 | 7,194,344 | 11,118,172 | 11,021,546 | 81.3 |
| 329T | 289.4 | 130,130,396 | 86,206,118 | 6,717,117 | 6,559,905 | 15,245,022 | 15,402,234 | 76.4 |
| 330T | 307.1 | 115,186,736 | 76,685,504 | 7,395,963 | 8,539,513 | 11,854,653 | 10,711,103 | 80.4 |
| 332T | 318.4 | 128,013,194 | 86,372,508 | 8,652,518 | 9,379,376 | 12,167,825 | 11,440,967 | 81.6 |
| 333T | 276.5 | 120,571,262 | 85,355,572 | 5,245,765 | 4,459,207 | 12,362,080 | 13,148,638 | 78.8 |
| 334T | 288.7 | 141,156,764 | 90,091,204 | 7,904,719 | 9,476,349 | 17,628,061 | 16,056,431 | 76.1 |
| 335T | 305.4 | 121,053,556 | 82,890,376 | 7,158,899 | 6,748,293 | 11,922,691 | 12,333,297 | 80.0 |
| 336T | 280.9 | 136,104,548 | 94,891,864 | 5,929,519 | 6,331,455 | 14,676,823 | 14,274,887 | 78.7 |
| 337T | 277.8 | 114,275,332 | 71,484,690 | 6,114,925 | 6,362,597 | 15,280,396 | 15,032,724 | 73.5 |
| 339T | 319.3 | 90,043,964 | 66,434,492 | 4,801,036 | 5,179,277 | 7,003,700 | 6,625,459 | 84.9 |
| 342T | 279.9 | 125,943,706 | 94,479,594 | 5,443,544 | 5,238,161 | 10,288,512 | 10,493,895 | 83.5 |
| 345T | 221.5 | 160,037,192 | 78,038,730 | 3,611,235 | 3,308,971 | 37,387,996 | 37,690,260 | 53.1 |
| 346T | 278.6 | 122,171,478 | 80,889,118 | 5,521,154 | 5,304,949 | 15,120,026 | 15,336,231 | 75.1 |
| 347T | 289.0 | 183,556,118 | 135,744,332 | 8,479,422 | 7,043,590 | 15,426,471 | 16,862,303 | 82.4 |
| 348T | 292.4 | 140,028,012 | 96,810,784 | 6,838,516 | 6,470,826 | 14,770,098 | 15,137,788 | 78.6 |
| 349T | 302.6 | 132,346,440 | 96,218,850 | 4,903,215 | 4,203,000 | 13,160,580 | 13,860,795 | 79.6 |
| 350T | 301.0 | 155,210,930 | 116,255,240 | 8,575,497 | 6,064,931 | 10,902,348 | 13,412,914 | 84.3 |
| 351T | 274.8 | 161,659,948 | 105,870,014 | 6,007,652 | 4,659,793 | 21,887,315 | 23,235,174 | 72.1 |
| 352T | 305.4 | 149,131,472 | 98,306,408 | 9,924,161 | 9,817,026 | 15,488,371 | 15,595,506 | 79.2 |
| 353T | 292.6 | 142,526,836 | 87,129,104 | 9,780,229 | 9,875,573 | 17,918,637 | 17,823,293 | 74.9 |
| 354T | 273.4 | 208,222,196 | 110,288,890 | 9,638,332 | 9,801,276 | 39,328,321 | 39,165,377 | 62.3 |
| 355T | 312.7 | 163,850,950 | 113,053,420 | 11,872,452 | 8,829,701 | 13,526,313 | 16,569,064 | 81.6 |
| 356T | 305.7 | 168,371,470 | 100,825,628 | 11,560,995 | 11,905,285 | 22,211,926 | 21,867,636 | 73.8 |
| 357T | 298.6 | 162,044,476 | 120,180,400 | 6,386,355 | 7,130,575 | 14,545,683 | 13,801,463 | 82.5 |
| 358T | 308.6 | 160,844,052 | 122,355,920 | 7,846,444 | 6,863,406 | 11,397,622 | 12,380,660 | 85.2 |
| 359T | 326.5 | 124,639,188 | 92,995,446 | 6,841,521 | 6,523,386 | 8,980,350 | 9,298,485 | 85.3 |
| 360T | 335.3 | 139,466,566 | 93,784,442 | 9,976,273 | 9,697,841 | 12,864,789 | 13,143,221 | 81.4 |
| 361T | 287.0 | 174,105,708 | 118,593,930 | 9,344,113 | 6,704,133 | 18,411,776 | 21,051,756 | 77.3 |
| 362T | 312.9 | 154,331,870 | 105,562,122 | 10,899,596 | 10,740,248 | 13,485,278 | 13,644,626 | 82.4 |
| 363T | 289.6 | 212,427,826 | 140,624,028 | 10,819,014 | 12,003,380 | 25,082,885 | 23,898,519 | 76.9 |
| 301N* | 310.1 | 196,437,198 | 99,325,506 | 14,756,135 | 18,012,072 | 33,799,711 | 30,543,774 | 67.2 |
| 302N* | 325.3 | 162,226,016 | 103,672,018 | 10,296,129 | 13,156,839 | 18,980,870 | 16,120,160 | 78.4 |
| 355N | 324.0 | 244,596,524 | 180,065,396 | 14,547,275 | 13,090,679 | 17,718,289 | 19,174,885 | 84.9 |
| 356N | 321.9 | 127,410,204 | 94,838,432 | 7,047,113 | 6,136,311 | 9,238,773 | 10,149,575 | 84.8 |
| 353N | 327.8 | 238,862,882 | 141,342,310 | 24,038,441 | 17,326,174 | 24,721,845 | 31,434,112 | 76.5 |

301N* is 2 adjacent samples pooled together: 304N, 309N. 302N* is 3 adjacent samples pooled together: 316N, 319N, 323N.
