# Supplementary material for: Identification of HBV-MLL4 Integration and Its Molecular Basis in Chinese Hepatocellular Carcinoma
Source: PLoS One. 2015 Apr 22;10(4):e0123175. doi: 10.1371/journal.pone.0123175 (PMC4406717; doi:10.1371/journal.pone.0123175)
Supplement: S2 Table — (DOCX) [file pone.0123175.s004.docx]

**S2** **Table. All HBV-human fusions detected.**

| **Sample** | **#**  **reads** | **HBV**  **position** | **Host**  **chr** | **Host**  **position** | **Human**  **Gene** | **Human**  **Exon** | **Fusion Junction Sequence** |
| --- | --- | --- | --- | --- | --- | --- | --- |
| 302N* | 53 | 1813 | 15 | 91509760 | PRC1 | 14 | TAGGCATAAATTGGTCTGTTCACCAGCACCgtgtaacagaactgaacacaatttaacaaa |
| 302N* | 10 | 1796 | 15 | 91509751 | PRC1 | 14 | TTTGTACTAGGAGGCTGTAGGCATAAATTGgccctttgttgggggtatgcatgtatgaac |
| 355N* | 7 | 1869 | 10 | 102723937 | FAM178A | 20 | CCCAAAGCCACCCAAGGCACAGCTTGGAGGgtagagagggagaaatgttcattgcacaga |
| 355N* | 5 | 1784 | 1 | 151032547 | MLLT11 | 1 | TGGAAAAATACTCTGATTCTTAAAAATACTtacagcctcctagtacaaagatcatta |
| 355N* | 3 | 464 | 7 | 74125369 | GTF2I | 9 | AGAAAGGCCTTGTAAGTTGGCGAGAAAGTGtcacatgcgatctacattaatatcaagtct |
| 355N* | 3 | 458 | 2 | 216253024 | FN1 | 26 | TCACTACATATTCTGTACCAGGCAGGAGATcttggtagtccagaagaaccaacaagaaga |
| 314T | 41 | 456 | 17 | 10401079 | MYH1 | 31 | CTTCTTGTTGGTTCTTCTGGACTACCAAGGgcggcacaggcagcatttgtcctctcaaca |
| 314T | 3 | 343 | 19 | 30313234 | CCNE1 | 10 | CTGCTTCGGCCTTGTATCATTTCTCGTCATctcttgtcctccaatttgtcctggctatcg |
| 314T | 5 | 2087 | 19 | 33517538 | RHPN2 | 3 | CTCAGGCAAGCTATTCTGTGTTgggagtggccacaaactcaaaggtgcagga |
| 314T | 3 | 2062 | 19 | 33535159 | RHPN2 | 2 | ACATTGTTCACCTCACCATACAGCACTCAGagtggccacaaactcaaagg |
| 315T | 71 | 294 | 19 | 36213551 | MLL4 | 5 | GGGACTGCGAATTTTGGCCAGGACACGTGGcccgggcctgacccagggccacagcagcat |
| 315T | 81 | 1825 | 19 | 36213556 | MLL4 | 5 | GGTCTGTTCACCAGCACCATGCAACTTTTTgcctgaagatgtccctcgcctcagtgccct |
| 316T | 77 | 1824 | 19 | 36212238 | MLL4 | 3 | TGGTCTGTTCACCAGCACCATGCAACTTTTatcggtgcttactcctcctcctcttggggc |
| 320T | 4 | 2934 | 19 | 36212705 | MLL4 | 3 | GCAGCAGCAGAAGGTGGCAGCTTCCATGCCgttggacccagcgttcggagccaactcaaa |
| 322T | 3 | 323 | 13 | 45914856 | TPT1 | 2 | CCTTCTGTCCTACTGACCATCTTCCCCTCCacctccaatcactcaccaacctcttgtccc |
| 323T | 3 | 458 | 6 | 84320396 | SNAP91 | 11 | TCTTCTTGTTGGTTCTTCTGGACTACCAAGatctggtgctccctctccattaagtaagtc |
| 325T | 23 | 1824 | 10 | 12070978 | UPF2 | 3 | TGGTCTGTTCACCAGCACCATGCAACTTTTtgagtgtgggactcccgatcagcattaata |
| 328T | 62 | 3139 | 19 | 36212607 | MLL4 | 3 | GGGAGTAGGCTGTCTTCCTGACTGCCGATTagacagcggcaaggaacccacgcccgcaat |
| 330T | 83 | 1807 | 2 | 43902713 | LOC728819 | 1 | AGGCTGTAGGCATAAATTGGTCTGTTCACCagcttattgaagaggcattgtctaataacc |
| 335T | 3 | 1828 | 19 | 12841836 | C19orf43 | 3 | TATCATCGTCACCGCACTGGTGAGCTTTGTactttttcacctctgcctaatcatctcatg |
| 335T | 10 | 1551 | 1 | 161193408 | APOA2 | 1 | GGCGGCCTAGCCAGCGTCTCTGTCCTTGGTgtctgtgccttctcatctgccggaccgtgt |
| 335T | 7 | 422 | 1 | 161193413 | APOA2 | 1 | CCAGCGTCTCTGTCCTTGGTGTCTGTGCCTcatcttcttgttggttcttctggactacca |
| 335T | 7 | 1109 | 3 | 186338806 | AHSG | 7 | GATATTGTTTACACAGAAGGGCCTTGTctgctggccacgcaagtgtcacatgcgatc |
| 335T | 3 | 1526 | 9 | 116838918 | AMBP | 2 | GTGCTGGTCATGCTGATCTCCGCCTCTGTAgcgcacctctctttacgcggtctccccgtc |
| 348T | 96 | 2103 | 19 | 36212560 | MLL4 | 3 | CTTCCAAATTACTTCCCACCCAGGTGGCCAgaggcatctgctgtggtgacggcggtggct |
| 351T | 5 | 1985 | 19 | 36212705 | MLL4 | 3 | ATACAGAGCAGAGGCGGTGTCGAGGAGATCggcatggaagctgccaccttctgctgctgc |
| 351T | 3 | 281 | 19 | 36212705 | MLL4 | 3 | GCAGCAGCAGAAGGTGGCAGCTTCCATGCCgggggagcacccacgtgtcctggccaaaat |
| 353T | 50 | 2297 | 19 | 36213611 | MLL4 | 5 | TGTTGATAAGATAGGGGCATTTGGTGGTCTcgaggtcctgccgatcccggagagggaggg |
| 353T | 6 | 1826 | 19 | 36213620 | MLL4 | 5 | TTCACCAGCACCATGCAACTTTTTCgatacatcatcggcgtccgagactgagagt |
| 355T | 13 | 2449 | 12 | 116534555 | MED13L | 4 | AGAAGATCTCAATCTCGGGAATCTCAATGTgcctaatggataagaacttcgttaggattg |
| 358T | 18 | 2298 | 19 | 36212705 | MLL4 | 3 | GTGTTGATAAGATAGGGGCATTTGGTGGTCggcatggaagctgccaccttctgctgctgc |
| 358T | 4 | 457 | 17 | 13933678 | COX10-AS1 | 3 | TCTTCTTGTTGGTTCTTCTGGACTACCAAGccatccatgtaagacatgacttgctg |
| 360T | 3 | 1782 | 19 | 8386583 | RPS28 | 2 | CTGGGCAGGACCGGTTCTCAGGGACAGTGCacagcctcctagtacaaagatcattaacct |

* Tumor adjacent tissues
