# Supplementary material for: Identification of HBV-MLL4 Integration and Its Molecular Basis in Chinese Hepatocellular Carcinoma
Source: PLoS One. 2015 Apr 22;10(4):e0123175. doi: 10.1371/journal.pone.0123175 (PMC4406717; doi:10.1371/journal.pone.0123175)
Supplement: S3 Table — (DOCX) [file pone.0123175.s005.docx]

**S3 Table. MLL4 gene copy number gain in HBV positive samples.**

| **HBV status** | **subtype** | **MLL4 GCN gain** | **NO**  **MLL4 GCN gain** | **Total** |
| --- | --- | --- | --- | --- |
| HBV+ | Total | 50 | 8 | 58 |
|  | HCC HBV C type | 28 | 6 |  |
|  | HCC HBV B type | 8 | 2 |  |
|  | Adjacent | 14 | 0 |  |
| HBV- | Total | 3 | 3 | 6 |

*p*=0.058, OR=6.0
