# Supplementary material for: Identification of HBV-MLL4 Integration and Its Molecular Basis in Chinese Hepatocellular Carcinoma
Source: PLoS One. 2015 Apr 22;10(4):e0123175. doi: 10.1371/journal.pone.0123175 (PMC4406717; doi:10.1371/journal.pone.0123175)
Supplement: S4 Table — . (DOCX) [file pone.0123175.s006.docx]

**S4 Table. cDNA sequences of HBV-MLL4 junction sites.**

| **Junction sites** | **Primer F (5'-3')** | **Primer R (5'-3')** | **PCR products** | **Sanger sequencing results** |
| --- | --- | --- | --- | --- |
| 315T UP | GGTCTGAAGATGAGTCGGTGGAAGC | ttggaggacaagaggttggtgagtg | 1 (Long) | Confirmed RNASeq junction site, followed by intron 4 in cDNA |
|  |  |  | 2 (Short) | Confirmed RNASeq junction site |
| 315T DN | ctgttcaccagcaccatgcaactt | GACCGTGACGGGACACTCTCAGT | 1 (Long) | Confirmed RNASeq junction site, followed by intron 5 in cDNA |
|  |  |  | 2 (Short) | Confirmed RNASeq junction site |
| 316T DN | caacgaccgaccttgaggcatactt | ATCGAGGCAGGCTGAGGTGGTT | 1 | Confirmed RNASeq junction site |
| 320T UP | CGCCGTCACCACAGCAGATG | actgcatggcctgaggatgactgt | 1 (Long) | Junction site consistent with gDNA |
|  |  |  | 2 (Short) | Confirmed RNASeq junction site |
| 328T UP | GGCCACAGACACAGGCTCAGCTACT | actgcatggcctgaggatgactgt | 1 | Confirmed RNASeq junction site |
| 348T UP | ACCACTCCTGTTAAGGCCGAGGTGT | cctggatgctgggtcttccaaatta | 1 | Confirmed RNASeq junction site |
| 351T-1 UP | ACTACCGCCACCACAGCCACAG | ctaaggcctcccgatacagagcaga | 1(Long) | Junction site consistent with gDNA |
|  |  |  | 2(Short) | Confirmed RNASeq junction site |
| 351T-2 UP | ACTACCGCCACCACAGCCACAG | gaggttggggactgcgaattttg | 1 (Long | Confirmed RNASeq 351T-1 junction site |
|  |  |  | 2 (Middle) | Confirmed RNASeq 351T-1 junction site, but followed by deletion of 477bp |
|  |  |  | 3 (Short) | Confirmed RNASeq junction site |
| 353T UP | TCCTCAAGAGAGCCAAAGTGCAGC | cctgcctcgtcgtctaacaacagta | 1 | Confirmed RNASeq junction site |
| 353T DN | caacgaccgaccttgaggcatactt | CAGAACCTGTGGGCTCTGATTCTCC | 1(Long) | Junction site consistent with gDNA |
|  |  |  | 2(Short) | Contain 2 products, one confirmed RNASeq results. The other followed by 111bp deletion after junction site. |
| 358T UP | TCCTCAAGAGAGCCAAAGTGCAGC | cctgcctcgtcgtctaacaacagta | 1 (Long) | Junction site consistent with gDNA |
|  |  |  | 2 (Short) | Confirmed RNASeq junction site |
| GAPDH | GAAGGTGAAGGTCGGAGTC | GAAGATGGTGATGGGATTTC |  |  |
