# Supplementary material for: Identification of HBV-MLL4 Integration and Its Molecular Basis in Chinese Hepatocellular Carcinoma
Source: PLoS One. 2015 Apr 22;10(4):e0123175. doi: 10.1371/journal.pone.0123175 (PMC4406717; doi:10.1371/journal.pone.0123175)
Supplement: S5 Table — (DOCX) [file pone.0123175.s007.docx]

**S5 Table. gDNA and cDNA structure of HBV-MLL4 integration.**

| **Sample** | **Genomic structure** | | | | | **Downstream cDNA structure** | | | | | | |
| --- | --- | --- | --- | --- | --- | --- | --- | --- | --- | --- | --- | --- |
|  | **HBV** | **MLL4 up-stream** | **MLL4 down-stream** | **MLL4 position** | **MLL4 loss** | **Transcripts** | | **HBV insertion** | **MLL4 junction site** | **MLL4 position** | **HBx-MLL4 Frame** | **HBc-MLL4 Frame** |
| 315T | 294-1825 | 36213551 | 36213556 | exon 5 | 4bp | T1 | 294 -1825 | | 36213556 | exon 5 | in frame | frame shift |
| 316T | 2700-1824 | 36212237 | 36212238 | exon 3 | ins31polyA | T1 | 2700 -1824 | | 36212238 | exon 3 | frame shift | in frame |
| 348T | 2103-1809 | 36212560 | 36212576 | exon 3 | 15bp insG | T1 | 1374-1809 | | 36212576 | exon 3 | frame shift | / |
| 351T | 1821-1783 | 36213184 | 36213196 | intron3 | 11bp | T1 | 1374 -1783 | | 36213196 | intron 3 | frame shift | / |
| 353T | 2297-1826 | 36213611 | 36213620 | exon5-intron5 junction | 8bp | T1 | 1374 -1826 | | 36213620 | intron 5 | frame shift | frame shift |
|  |  |  |  |  |  | T2 | 1374 -1716 | | 36213897 | exon6 | in frame | / |
| 358T | 2295-1822 | 36212901 | 36212934 | intron3 | 32bp | T1 | 1374 -1822 | | 36212934 | intron3 | frame shift | frame shift |
|  |  |  |  |  |  | T2 | 1374 -1700 | | 36213261 | exon4 | in frame | / |
|  |  |  |  |  |  | T3 | 1374 -1646 | | 36213261 | exon4 | in frame | / |
|  |  |  |  |  |  | T4 | 1374 -1634 | | 36213261 | exon4 | in frame | / |
| 320T | 2717-1823 | 36212841 | 36212935 | intron3 | 93bp | T1 | 1374 -1823 | | 36212935 | intron 3 | frame shift | frame shift |
|  |  |  |  |  |  | T2 | 1374-1646 | | 36213261 | exon4 | in frame | / |
|  |  |  |  |  |  | T3 | 1374-1634 | | 36213261 | exon4 | in frame | / |
| 328T | 3139-1801 | 36212607 | 36212770 | exon3-intron3 | 162bp | T1 | 3139-458 | | 36213261 | exon4 | / | / |
|  |  |  |  |  |  | T2 | 1374-1801 | | 36212770 | intron3 | frame shift | / |
|  |  |  |  |  |  | T3 | 1374-1646 | | 36213261 | exon4 | in frame | / |
|  |  |  |  |  |  | T4 | 1374-1634 | | 36213261 | exon4 | in frame | / |
