# Supplementary material for: Identification of HBV-MLL4 Integration and Its Molecular Basis in Chinese Hepatocellular Carcinoma
Source: PLoS One. 2015 Apr 22;10(4):e0123175. doi: 10.1371/journal.pone.0123175 (PMC4406717; doi:10.1371/journal.pone.0123175)
Supplement: S6 Table — (DOCX) [file pone.0123175.s008.docx]

**S6 Table. Primer sequences used for determining gDNA and cDNA of HBV-MLL4 integration**

| **DNA/cDNA** | **Sample name** | | **Primer F (5'-3')** | **Primer R (5'-3')** |
| --- | --- | --- | --- | --- |
| DNA | 315T | | GGTCTGAAGATGAGTCGGTGGAAGC | ACCGTGACGGGACACTCTCAGTCT |
|  | 316T | | ACATTTCGCTGGACCTCACTGACC | AGTCATCGGCAGGAGGAGGCTCT |
|  | 320T | | CGCCGTCACCACAGCAGATG | GACCGTGACGGGACACTCTCAGT |
|  |  |  | ccaatcactcaccaacctcttgtcc | GACCGTGACGGGACACTCTCAGT |
|  | 328T | | CGCCGTCACCACAGCAGATG | GACCGTGACGGGACACTCTCAGT |
|  | 348T | | GGCCACAGACACAGGCTCAGCTACT | cctggatgctgggtcttccaaatta |
|  |  |  | ttggaagacccagcatccaggg | GACCGTGACGGGACACTCTCAGT |
|  | 351T | 351T-1 | TCCTCAAGAGAGCCAAAGTGCAGC | cctggatgctgggtcttccaaatta |
|  |  |  | gagtctccggaacattgttcacctc | GACCGTGACGGGACACTCTCAGT |
|  |  | 351T-2 | ACTACCGCCACCACAGCCACAG | atgataaaacgccgcagacacatcc |
|  |  |  | ccaatcactcaccaacctcttgtcc | GACCGTGACGGGACACTCTCAGT |
|  | 353T |  | GGTCTGAAGATGAGTCGGTGGAAGC | GACCGTGACGGGACACTCTCAGT |
|  | 358T | | ACTACCGCCACCACAGCCACAG | cggaagtgttgataagataggggcatt |
|  |  |  | ctactgttgttagacgacgaggcagg | GACCGTGACGGGACACTCTCAGT |
| cDNA | 315T, 320T, 328T, 348T, 351T, 353T, 358T | | CGCCGTCACCACAGCAGATG | GACCGTGACGGGACACTCTCAGT |
|  | 316T | | ACATTTCGCTGGACCTCACTGACC | AGTCATCGGCAGGAGGAGGCTCT |
| cDNA  (HBx-MLL4) | All 8 samples | | ATGGCTGCTAGGCTGTGCTGC | GACCGTGACGGGACACTCTCAGT |
